# Supplementary material for: Block Copolymers of Poly(N-Vinyl Pyrrolidone) and Poly(Vinyl Esters) Bearing n-alkyl Side Groups via Reversible Addition-Fragmentation Chain-Transfer Polymerization: Synthesis, Characterization, and Thermal Properties
Source: Polymers (Basel). 2024 Aug 29;16(17):2447. doi: 10.3390/polym16172447 (PMC11398064; doi:10.3390/polym16172447)
Supplement: Supplementary file 1 [file polymers-16-02447-s001.zip › polymers-3173587-supplementary.pdf]

polymers

MDPI

Article

Block Copolymers of Poly(N-vinyl Pyrrolidone) and Poly(Vinyl Esters) bearing n-alkyl side-groups via RAFT polymerization.

Synthesis, Characterization and Thermal Properties

Nikoletta Roka, Theodosia-Panagiota Papazoglou and Marinos Pitsikalis*

Industrial Chemistry Laboratory, Department of Chemistry, National and Kapodistrian University of Athens, Panepistimiopolis Zografou, 15771 Athens Greece

* Correspondence: pitsikalis@chem.uoa.gr

Supporting Information Section

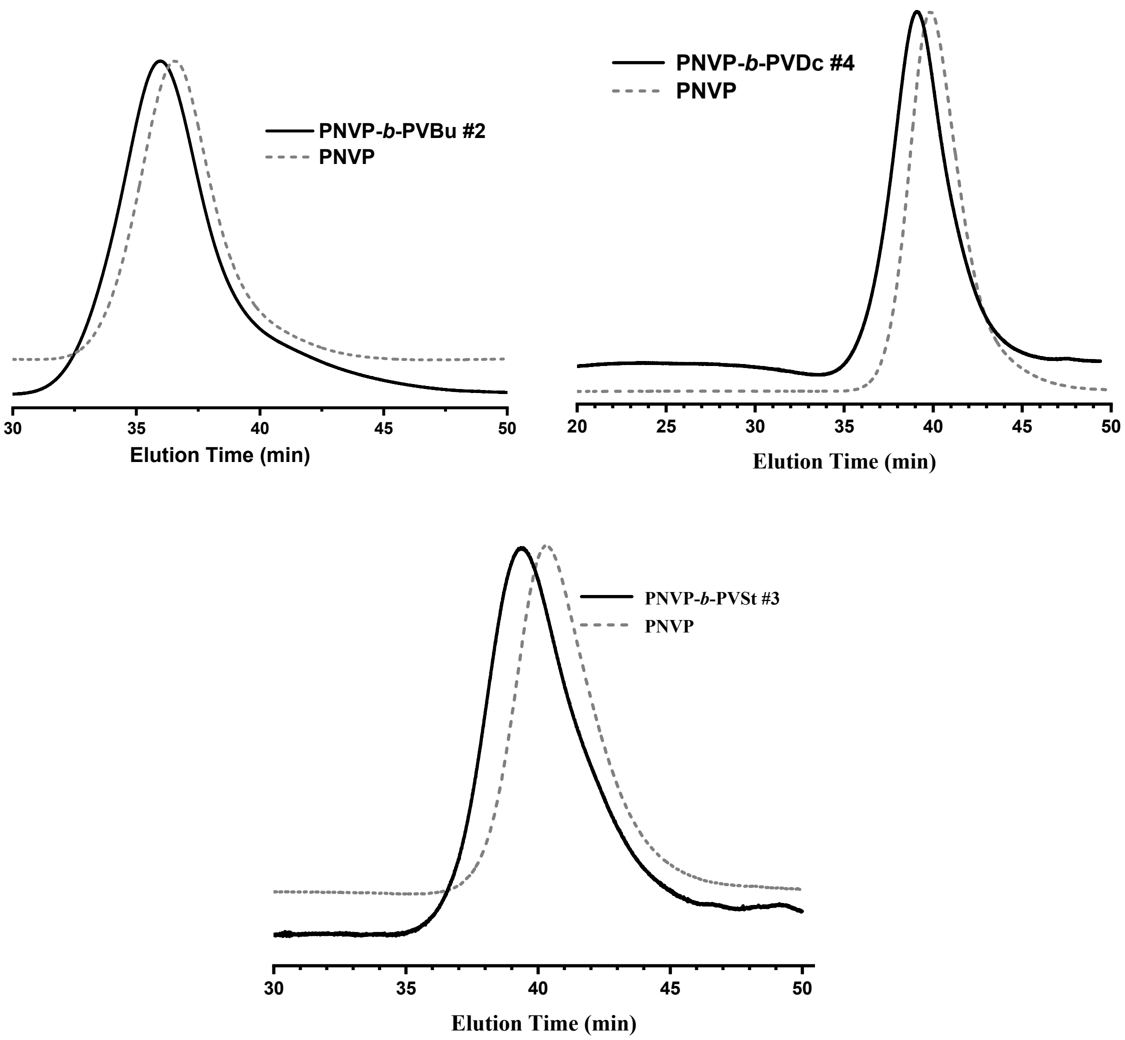

Figure S1. SEC traces from the synthesis of the block copolymer PNVP-*b*-PVBu #2, PNVP-*b*-PVDc #4 and PNVP-*b*-PVSt #3

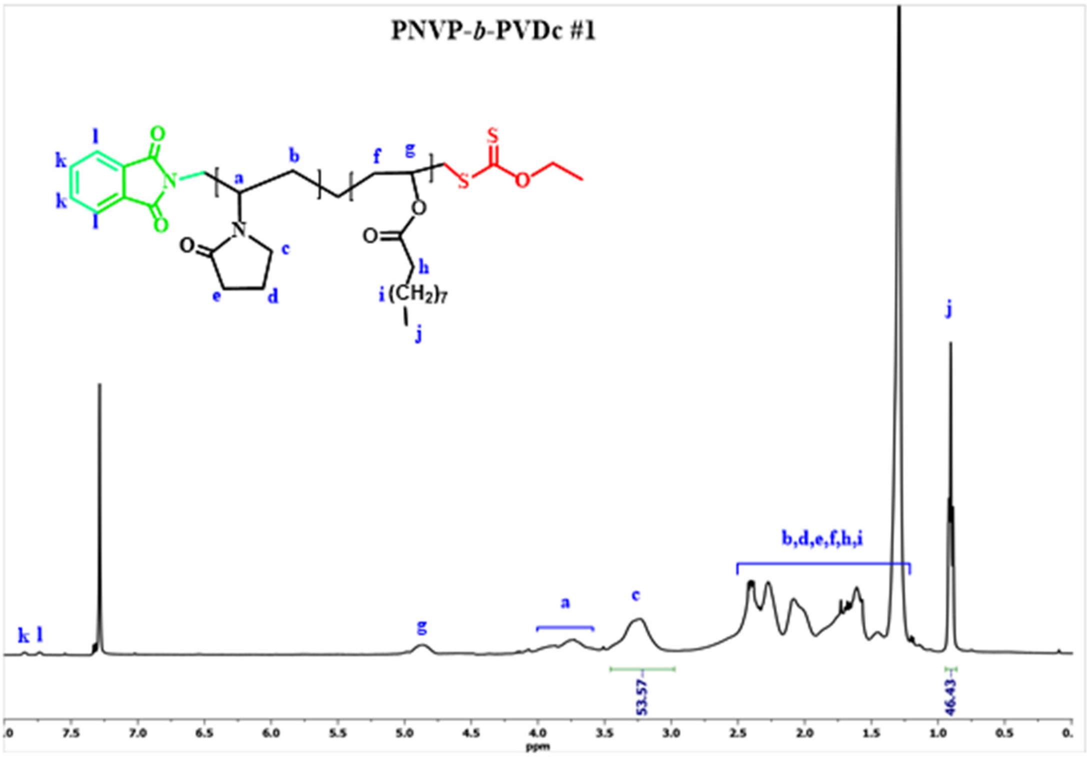

Figure S2. ^{1}H NMR spectrum of the block copolymer PNVP-b-PVDc #1

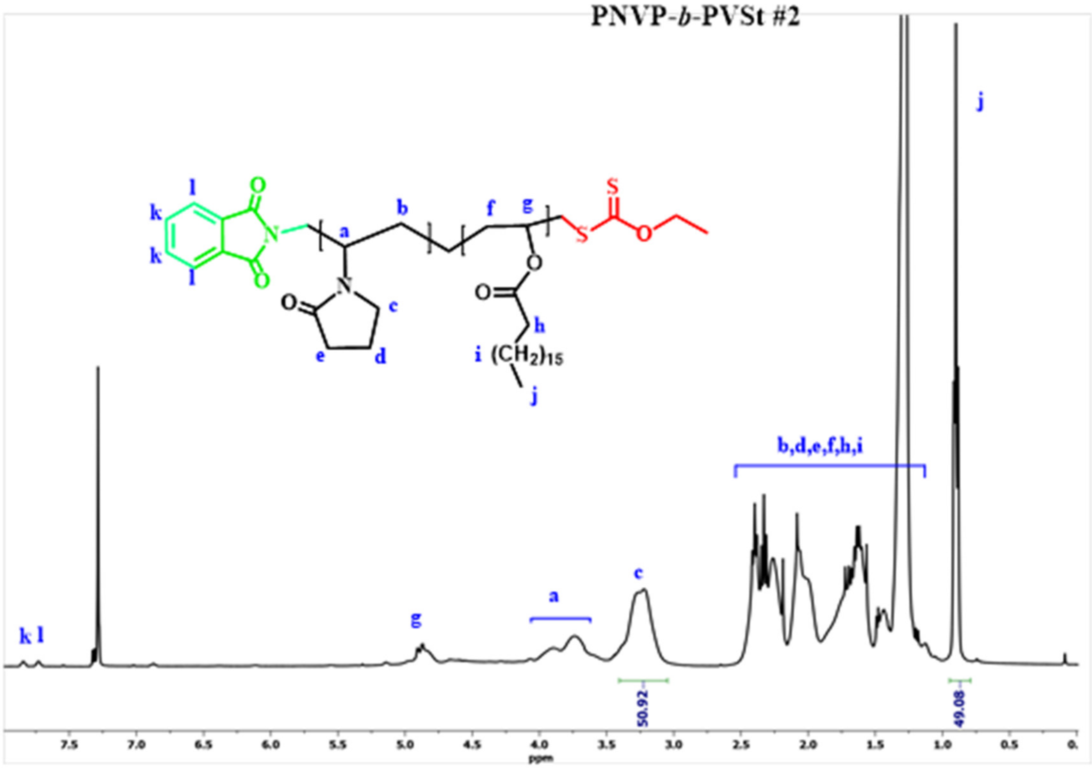

Figure S3. ^{1}H NMR spectrum of the block copolymer PNVP-b-PVSt #2

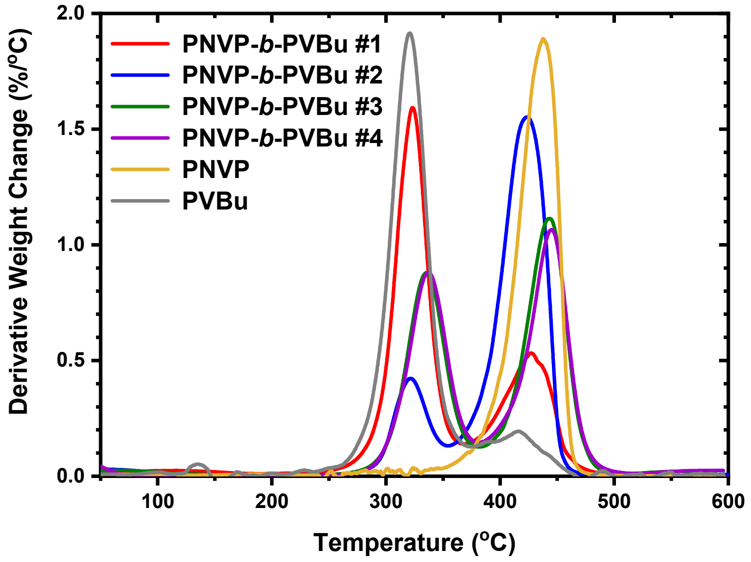

Figure S4. DTG plots of the PNVP-*b*-PVBu copolymers at 10° C/min

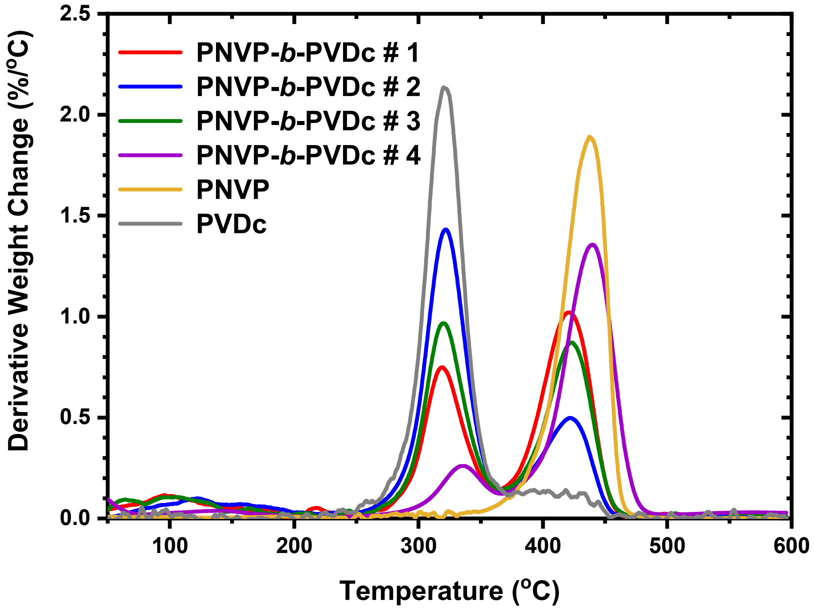

Figure S5. DTG plots of the PNVP-*b*-PVDc copolymers at 10° C/min

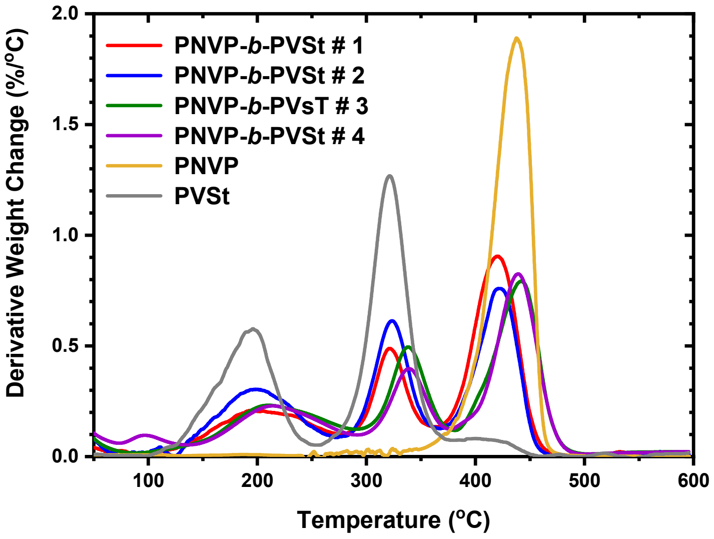

Figure S6. DTG plots of the PNVP-*b*-PVSt copolymers at 10° C/min
